# Endoscopic ultrasound-guided retrieval of a migrated plastic stent from a pelvic abscess

**DOI:** 10.1055/a-2419-1098

**Published:** 2024-10-14

**Authors:** Koichi Soga, Yuto Suzuki, Fuki Hayakawa, Takeshi Fujiwara, Yo Fujimoto, Ikuhiro Kobori, Masaya Tamano

**Affiliations:** 1 26263 Gastroenterology, Dokkyo Medical University Saitama Medical Center, Koshigaya, Japan

Endoscopic ultrasound (EUS)-guided pelvic abscess drainage is a minimally invasive procedure; however, the anatomical challenges and restricted space can lead to complications 1 . This case report describes the migration of a plastic stent into a pelvic abscess cavity and its subsequent retrieval using a thin endoscope in a 55-year-old Japanese man with a pelvic abscess secondary to a hepatic abscess.

One month prior to presentation, the patient underwent EUS-guided pelvic abscess drainage, during which the commercial plastic stent failed to detach. As an emergency solution, a self-made plastic stent (Flexima; Boston Scientific, Marlborough, Massachusetts, United States) was inserted 2 3 4 , which led to improvement of the pelvic abscess ( Fig. 1 ). Abdominal computed tomography and radiography performed in preparation for the removal of the self-made plastic stent revealed that the stent had rotated several times and lodged within the pelvic abscess cavity ( Fig. 2 ). EUS-guided pelvic abscess drainage was used to retrieve the migrated stent ( Video 1 ). The residual pelvic abscess cavity and self-made plastic stent were located using EUS. A 19-G needle was used to puncture the pelvic abscess, followed by placement of a guidewire into the cavity. The guidewire was left in place as the EUS endoscope was removed. A standard 9.9-mm endoscope (GIF-H290Z; Olympus, Tokyo, Japan) and an 8-mm dilation balloon were used to enlarge the puncture site. A 5.8-mm thin endoscope (GIF-1200N; Olympus) was then inserted, providing access to the pelvic abscess cavity through the dilation tract. Sufficient space and the migrated self-made plastic stent were identified within the cavity ( Fig. 3 ), and the stent was successfully retrieved using a 1.8-mm snare (SD-221L-25; Olympus) ( Fig. 4 ).

**Fig. 1:**
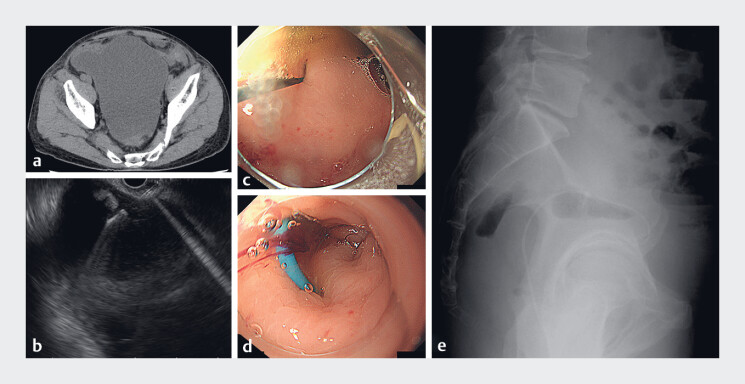
Initial endoscopic ultrasound (EUS)-guided pelvic abscess drainage. **a** Abdominal computed tomography revealed a pelvic abscess secondary to a hepatic abscess in the retroperitoneum. **b** EUS-guided pelvic abscess drainage was performed using a convex endoscope (UCT-260; Olympus, Tokyo, Japan). The abscess was punctured using a 19-G needle. **c–d** A commercial plastic stent (Piglet; Olympus) could not be released ( **c** ); therefore, a self-made plastic stent (Flexima; Boston Scientific, Marlborough, Massachusetts, United States) was inserted using a direct-view endoscope (GIF-H260; Olympus) ( **d** ). **e** Radiography revealed improvement in the pelvic abscess.

**Fig. 2:**
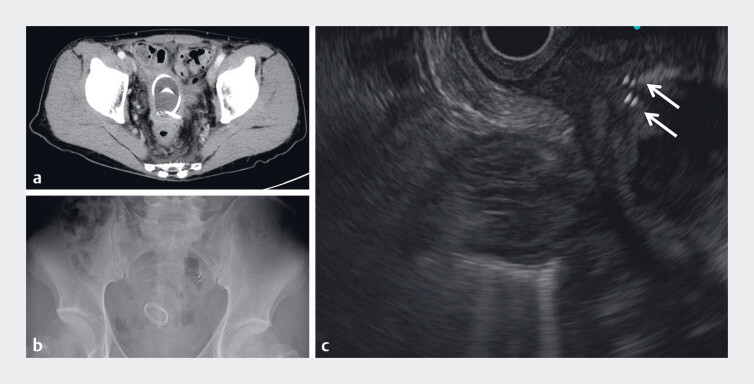
**a–b** Abdominal computed tomography ( **a** ) and radiography ( **b** ) revealed that the self-made plastic stent had rotated multiple times and lodged within the pelvic abscess cavity. **c** Endoscopic ultrasound showed the residual pelvic abscess cavity and migrated self-made plastic stent (arrows).

**Fig. 3:**
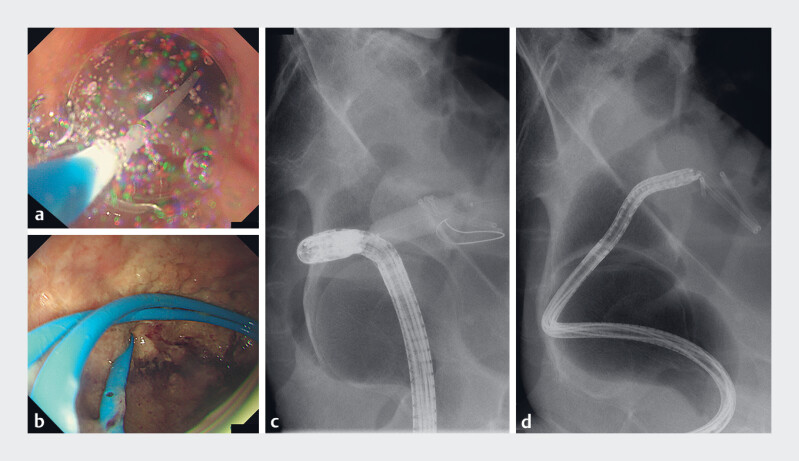
Retrieval of the migrated self-made plastic stent using an endoscopic ultrasound (EUS)-guided pelvic abscess drainage technique. **a** After the pelvic abscess was punctured using a 19-G needle, a guidewire was placed in the abscess cavity using a standard direct-view endoscope (9.9 mm; GIF-H290Z; Olympus, Tokyo, Japan), and the puncture tract was dilated using an 8-mm balloon. **b** A thin endoscope (5.8 mm; GIF-1200N; Olympus) was inserted to allow access to the abscess cavity through the dilation tract. Sufficient space and the migrated self-made plastic stent were detected within the pelvic abscess cavity. **c** Radiography revealed the guidewire within the pelvic abscess cavity. **d** The self-made plastic stent was successfully retrieved using a snare (SD-221L-25; Olympus).

**Fig. 4:**
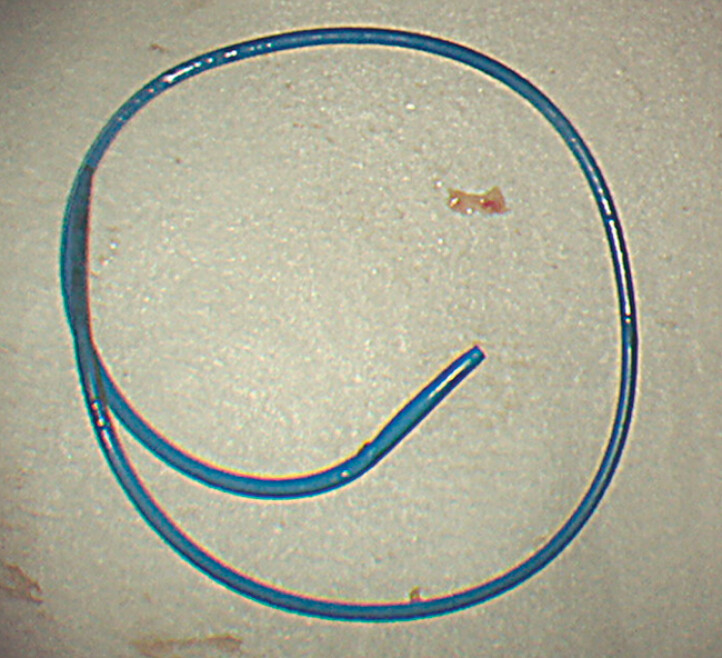
Successfully retrieved migrated plastic stent from a 55-year-old Japanese man with a pelvic abscess secondary to a hepatic abscess who underwent endoscopic ultrasound-guided pelvic abscess drainage.

Retrieval of a migrated plastic stent from a pelvic abscess using an endoscopic ultrasound-guided technique in a 55-year-old Japanese man.Video 1

The procedure was completed without complications, and the patient’s progress was favorable. Our troubleshooting approach, which applied our previously reported technique 5 , proved effective in this case. This highlights the importance of adopting adaptive strategies for managing unexpected events during interventional EUS procedures, based on prior experience and knowledge.

Endoscopy_UCTN_Code_CPL_1AK_2AD

## Notes

Endoscopy E-Videos https://eref.thieme.de/e-videos
*E-Videos* is an open access online section of the journal *Endoscopy* , reporting on interesting cases and new techniques in gastroenterological endoscopy. All papers include a high-quality video and are published with a Creative Commons CC-BY license. Endoscopy E-Videos qualify for HINARI discounts and waivers and eligibility is automatically checked during the submission process. We grant 100% waivers to articles whose corresponding authors are based in Group A countries and 50% waivers to those who are based in Group B countries as classified by Research4Life (see: https://www.research4life.org/access/eligibility/ ). This section has its own submission website at https://mc.manuscriptcentral.com/e-videos .
